# Microwave Attenuation of Graphene Modified Thermoplastic Poly(Butylene adipate-*co*-terephthalate) Nanocomposites

**DOI:** 10.3390/polym10060582

**Published:** 2018-05-25

**Authors:** Sima Kashi, S. Ali Hadigheh, Russell Varley

**Affiliations:** 1 Institute for Frontier Materials, Deakin University, Waurn Ponds, VIC 3216, Australia; russell.varley@deakin.edu.au; 2 School of Civil Engineering, Faculty of Engineering and IT, The University of Sydney, Sydney, NSW 2006, Australia; ali.hadigheh@sydney.edu.au

**Keywords:** graphene, nanocomposite, EMI shielding, frequency range, thickness range

## Abstract

With the widespread development and use of electronics and telecommunication devices, electromagnetic radiation has emerged as a new pollution. In this study, we fabricated flexible multifunctional nanocomposites by incorporating graphene nanoplatelets into a soft thermoplastic matrix and investigated its performance in attenuating electromagnetic radiation over frequency ranges of C (5.85–8.2 GHz), X (8.2–12.4 GHz), and Ku bands (12.4–18 GHz). Effects of nanofiller loading, sample thickness, and radiation frequency on the nanocomposites shielding effectiveness (SE) were investigated via experimental measurements and simulation. The highest rate of increase in SE was observed near percolation threshold of graphene. Comparison of reflectivity and absorptivity revealed that reflection played a major role in nanocomposites shielding potential for all frequencies while the low absorptivity was due to high power reflection at nanocomposite surface and thin thickness. Subsequently, effective absorbance calculations revealed the great potential of nanocomposites for absorbing microwaves, reaching more than 80%. Simulations confirmed the observed nanocomposites SE behaviours versus frequency. Depending on thickness, different frequency dependency behaviours were observed; for thin samples, SE remained unchanged, while for thicker samples it exhibited either increasing or decreasing trends with increasing frequency. At any fixed frequency, increasing the thickness resulted in sine-wave periodic changes in SE with a general increasing trend.

## 1. Introduction

In 1940, it was established theoretically that graphene is the building block of graphite [1], but it was not until 2004 that single layers of graphene were experimentally prepared in a laboratory [2]. As one of the thinnest materials in the universe [3], this new member of carbon allotropes has created tremendous excitement in materials research. Its exceptional properties include high thermal conductivity, superior mechanical properties and excellent electronic transport properties [4,5,6,7,8], providing the potential for graphene to be a promising material for a wide range of applications [9]. As examples, graphene could be used in new generations of high-speed and radio-frequency logic devices, electronic circuits, sensors, transparent and flexible electrodes for displays and solar cells, ultra-thin carbon films, and photodynamic and photothermal therapy [10,11,12,13,14].

One of the most significant areas in which graphene nanoplatelets have shown promising results is in reinforcing polymers to produce multi-functional nanocomposites with tunable properties [4,15,16,17,18]. Application of such hybrid materials for electromagnetic interference shielding has been the focus of many recent studies [19,20,21,22,23], as a light-weight replacement for the conventional metal-based shielding materials. Highly conductive metals have traditionally been used to attenuate undesirable electromagnetic radiation and protect sensitive devices [24]. However, as the world is moving towards smart, small and more functional electronic devices, electromagnetic interference (EMI) is emerging as new type of pollution, raising concerns for the proper function of highly sensitive electronics as well as human health [25,26,27,28,29]. As a result, polymeric nanocomposites with electromagnetic properties are attracting attention as the next generation of efficient, light-weight and flexible EMI shielding materials [26,30,31,32]. In the last few years, various polymeric matrices, including both thermoplastics and thermosets, have been embedded with graphene nanoplatelets at different concentrations, and the resultant nanocomposites have been examined for their efficiency in EMI attenuation: polyethylene [33], polyethyelene terephthalate [34], poly(dimethyl siloxane) [35], polymethylmethacrylate [36], polyaniline [37], poly(ethylene-vinyl acetate) [38], epoxy [39], polystyrene and epoxy resin [40], poly(dimethyl siloxane) [41]. Promising results have been reported on the EMI shielding performance of graphene-based nanocomposites. In a paper by Cao et al. [42], the advances in graphene-based EMI shielding materials, as well as the mechanisms of EMI shielding of graphene-based composite materials, were intensively reviewed. In another paper [43] the microwave response mechanism of graphene-dispersed systems, flexible graphene papers, graphene hybrids, and 3D graphene architectures were systematically reviewed. It was observed that such nanocomposites have the ability to perform well in harsh environments such as high temperatures up of to 200 °C [44,45]. Some recent studies have also shown that addition of small amounts of ferroferric oxide to composites, containing carbon nanofillers such as graphene [46] and carbon nanotubes [47], provides optimal synergistic effects of dielectric and magnetic loss, and therefore enhances the EMI shielding performance of the shielding material. It has also been reported that implanting small NiFe_2_O_4_ clusters on reduced graphene oxide can tune the magnetic properties of the composite and increase the shielding effectiveness of the shielding material [48].

In the current work, we studied the EMI shielding performance of a new graphene-based nanocomposite with poly(butylene adipate-*co*-terephthalate) (PBAT) as the matrix. This polymer, in spite of its origin (petroleum), is capable of complete biodegradation, depending on the composting environment. In a previous work [49], we investigated the microwave attenuation capability of biodegradable nanocomposites composed of graphene nanoplatelets (GNPs) and polylactide. In a later study [50], however, it was found that GNP nanocomposites with PBAT are thermally significantly more stable than nanocomposites with polylactide. In the present article, a wide range of microwave frequency, including C-, X-, and Ku-bands, is covered, rather than focusing only on the X-band frequency range. Furthermore, all PBAT/GNP nanocomposites were prepared with three different thicknesses so that the effect of thickness on the EMI shielding effectiveness of PBAT nanocomposite with varying GNP loadings could be examined experimentally and not just theoretically. Moreover, the SE behaviours of all PBAT/GNP nanocomposites were modelled as functions of both frequency and thickness over 8.2–12.4 GHz frequency range. Percolation behaviour of the GNPs in the polymeric matrix was also investigated with respect to both electrical conductivity and rheological behaviour.

## 2. Experimental

### 2.1. Materials and Nanocomposites Preparation

**Figure 1:**
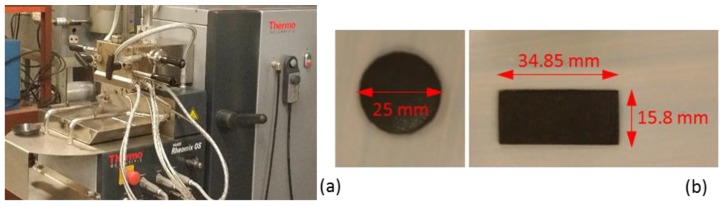
(**a**) Haake mixer and (**b**) rheology circular sample and C-band EMI shielding rectangular sample.

PBAT, under the catalogue name Ecoflex F Blend C1200, was purchased (BASF, Ludwigshafen, Germany). According to the technical data sheet, it has a density of 1.25–1.27 g/cm^3^ and melting range of 110–120 °C [51]. Grade M GNPs were obtained from XG Sciences (Lansing, MI, USA), exhibiting an average thickness of 6–8 nm, surface area of 120–150 m^2^/g, density of 2.2 g/cm^3^ and electrical conductivity of 10^2^ and 10^7^ S/m for perpendicular and parallel to the surface, respectively [52]. 60 g of PBAT pellets and GNPs were melt-mixed at different concentrations in the internal chamber of a Haake Rheomix OS R600 (Thermo Fisher Scientific, Waltham, MA, USA) (Figure 1a) using roller rotors at a temperature of 140 °C, which is approximately 20 °C above the melting range of PBAT, a mixing speed of 60 rpm and a mixing time of 10 min. The polymer pellets and the nanoplatelets were dried at 60 °C and 80 °C, respectively, overnight in convection ovens prior to melt-mixing. Even though PABT has a relatively high thermal stability [50], the pre-drying was carried out as a precaution to remove moisture and prevent possible degradation of the polymer during the mixing. The prepared nanocomposites were then moulded using a hot press at a temperature of 140 °C with a force of 80 kN for 5 min. The mixing ratios of GNPs in PBAT were 0, 3, 6, 9, 12, and 15 wt %, and nanocomposites with GNP volume contents of 0, 1.7, 3.5, 5.3, 7.2, and 9.1 vol % were obtained.

### 2.2. Characterisation

Pure PBAT and PBAT/GNP nanocomposites were snapped after exposure to liquid nitrogen. Surface morphology of the fractured samples was studied via scanning electron microscopy (Quanta 200 SEM, Field Electron and Ion Company, Hillsboro, OR, USA). Accelerated voltages of 5 kV and 30 kV were used for the polymeric and GNPs samples, respectively. DC conductivity measurements were carried out by using a HP 34420A nano-volt meter (Keysight Technologies, Santa Rosa, CA, USA) and a Keysight B2985A high resistance meter (Keysight Technologies, Santa Rosa, CA, USA). Both meters were used for the measurements in order to cover resistance values ranging from TΩ for samples with very low conductivity to kΩ for samples with higher conductivity. Dynamic rheology of the PBAT/GNP nanocomposites was assessed using a strain-controlled Advanced Rheometrics Expansion System (ARES) rheometer (Rheometric Scientific, Piscataway, NJ, USA) with a force transducer of torque range of 0.2–200 g·cm and parallel-plate fixture of 25 mm diameter (Figure 1b). Measurements were performed at 140 °C. Frequency (*ω*) sweeps were conducted over the range of 10^−2^–100 rad/s within the linear viscoelastic region of each sample (determined from strain sweeps).

**Figure 2:**
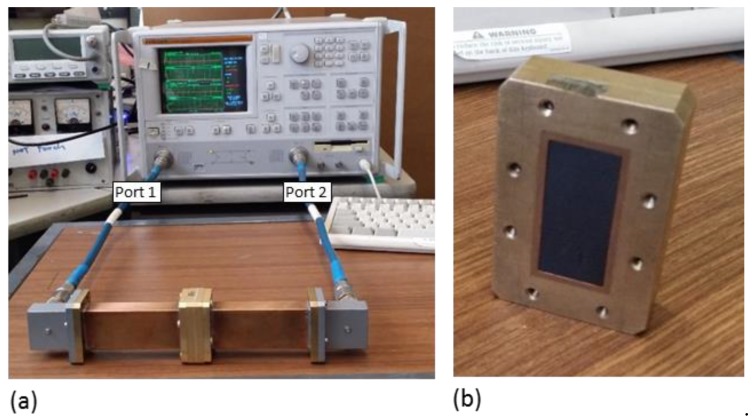
(**a**) Vector network analyser with C-band waveguide setup and (**b**) C-band sample holder with sample.

For EMI shielding measurement, a Wiltron vector network analyser (VNA) model 37269A was used to measure the scattering (S-) parameters of PBAT and PBAT/GNP nanocomposites over three frequency ranges of C-, X-, and Ku-bands, covering frequencies of 5.85–8.2 GHz, 8.2–12.4 GHz, and 12.4–18 GHz, respectively. Figure 2 shows the VNA with a WR-137 (C-band) waveguide and the C-band sample holder with a nanocomposite sample inside. Samples with thicknesses of 1, 1.5, and 2.8 mm were prepared for EMI shielding measurements using the compression moulding as explained in Section 2.1. Figure 1b demonstrates a sample prepared for C-band measurements. Once the EMI shielding performance of samples were measured for C-band, samples were cut into dimensions of 22.86 × 10.16 mm^2^ to fit the X-band sample holder for the WR-90 waveguide, and then into dimensions of 15.8 × 7.8 mm^2^ to fit the Ku-band sample holder for the WR-62 waveguide.

## 3. Results and Discussion

### 3.1. Morphology, Conductivity and Rheology

**Figure 3:**
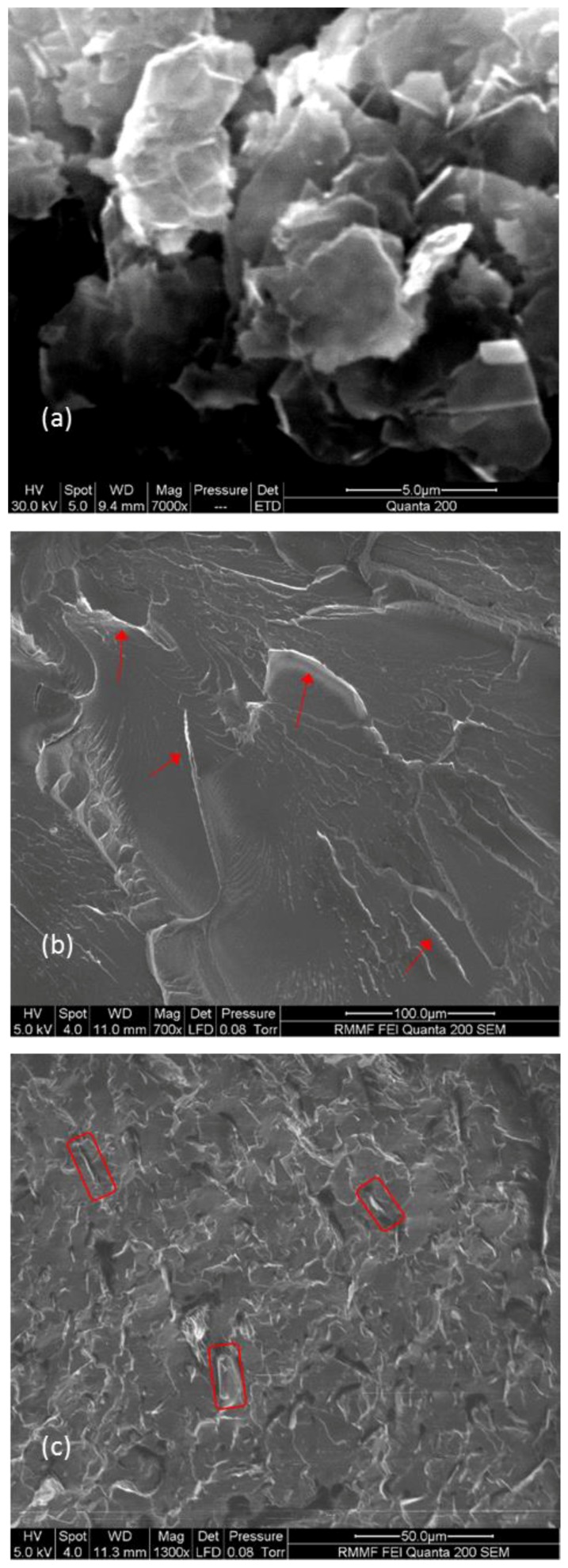
SEM images of (**a**) graphene nanoplatelets, (**b**) fractured cross section of virgin PBAT, and (**c**) fractured surface of PABT nanocomposite with 3 wt % GNPs.

Morphology of the PBAT/GNP nanocomposites and the state of GNP dispersion in the PBAT matrix were investigated via scanning electron microscopy. A high-magnification SEM image of the graphene nanoplatelets is depicted in Figure 3a, revealing flake-like shapes as well as a laminar structure for the platelets. Furthermore, the platelets are found to have a relatively smooth surface without cracks. Figure 3b shows the fractured surface of virgin PBAT formed after immersion in liquid nitrogen and then snapping the sample into two pieces. The surface morphology of PBAT nanocomposite with 3 wt % of GNPs is shown in Figure 3c, where the platelets appear as a brighter grey compared to the matrix, due to their higher conductivity. The brighter grey areas seen in Figure 3b for the PBAT sample with no GNPs, indicated by the red arrows, are sections with sharper edges compared to the smoother areas. The platelets in Figure 3c have a void surrounding, which is the GNP-matrix interphase, differentiating the platelets (light grey) from the sharp edges of the matrix. Morphology of PBAT/GNP nanocomposites and dispersion state of the nanoplatelets in the polymeric matrix have been studied in more detail in a report on the rheological behaviour of these nanocomposites [53].

**Figure 4:**
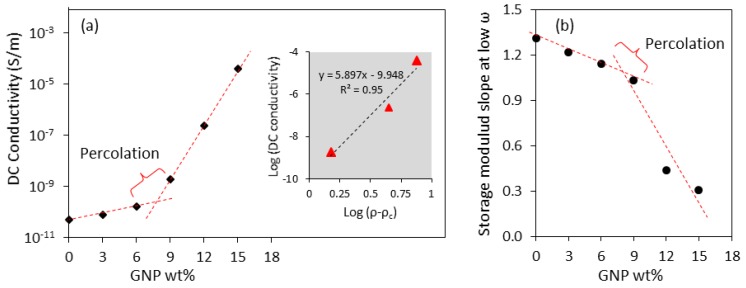
(**a**) Effect of GNP incorporation on the DC conductivity of PBAT and its electrical percolation, (**b**) rheological percolation of PBAT/GNP nanocomposites from dynamic frequency sweeps at 140 °C.

Incorporation of highly conductive nanoparticles such as GNPs in an insulating matrix like PBAT produces a hybrid material with tunable electrical conductivity, with the conductivity being dependent on the volume percent of the dispersed phase and the quality of dispersion. Figure 4a shows the effect of GNP loading on the DC electrical conductivity of PBAT. As can be seen, unmodified PBAT has a very low conductivity of about 10^−11^ S/m, and low concentrations of GNPs do not improve the conductivity of the system appreciably. This is due an encapsulation effect of the nanoplatelets by the matrix, preventing the formation of conductive pathways for the electrons within the system. However, when the concentration is raised above 6 wt % (3.5 vol %) a sudden increase in the conductivity of PBAT/GNP nanocomposites is observed. This can be attributed to the formation of an interconnected structure of GNPs within the matrix, that is to say, the percolation threshold, acting as electric charge carriers. Equation (1) describes the conductivity of a binary system with conductive filler loading of ρ, which is above its electrical percolation threshold $\rho_{c}$ [39,54,55,56]. In this equation, σ and α are the DC conductivity and the equation constant, respectively. The inset of Figure 4a illustrates the log *σ* − log (*ρ* − *ρ_c_*) plot for PBAT nanocomposites with GNP loadings of 9–15 wt %. With *ρ_c_* of 7.5 wt %, *α* and *t* are calculated to be 1.113 × 10^−10^ S/m and 5.897, respectively.

(1) $$\sigma =\alpha {\left(\rho -\rho_{c}\right)}^{t}.$$

The percolation threshold for a filled polymer is the filler loading at which the filler particles establish physical contact with one another and form a true network within the matrix [57]. This threshold is usually determined via rheological and conductivity measurements for fillers that are electrically conductive, as it is marked by a sudden change in these properties. As an example, Figure 4a shows such an increase in the DC conductivity of PBAT/GNP nanocomposites in the 6–9 wt % range, indicative of reaching the GNP percolation threshold. Figure 4b shows the changes in the slope of storage modulus obtained from rheological frequency sweeps of the PBAT/GNP nanocomposites at 140 °C. The change in this slope is indicative of the transition in rheological behaviour of the nanocomposites from liquid-like to solid-like, which is due to the presence of the nanoplatelets in the matrix. Rheological properties of PBAT/GNP nanocomposites have been comprehensively reported in previous studies [53,58,59].

### 3.2. EMI Shielding

**Figure 5:**
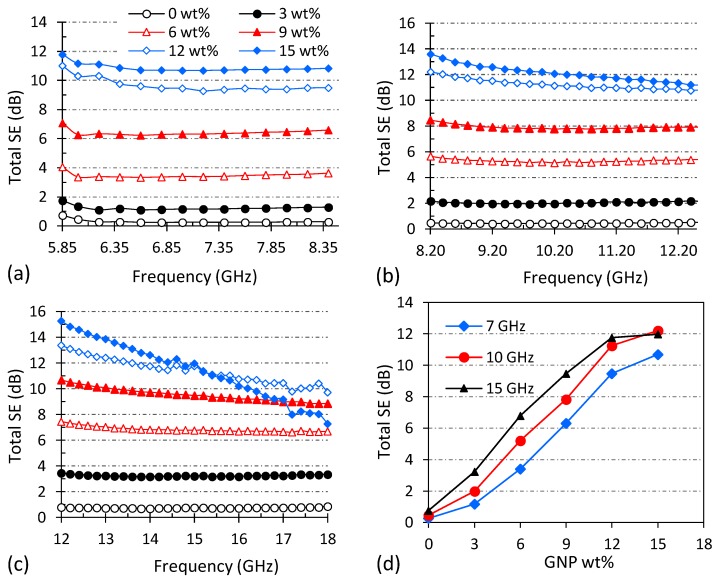
EMI shielding effectiveness of PBAT/GNP nanocomposites over (**a**) C-band, (**b**) X-band, (**c**) Ku-band, and (**d**) as a function of GNP loading at mid-range frequencies.

Figure 5 depicts the EMI shielding effectiveness (SE) profiles of 1 mm thick PBAT/GNP nanocomposites over the frequency ranges of 5.85–8.2 GHz, 8.2–12.4 GHz, and 12.4–18 GHz. It is worth mentioning here that since WR137, WR90, and WR62 waveguides were used for C-, X-, and Ku-bands, respectively, the SE of the nanocomposites for these three frequency ranges are demonstrated in different graphs. The EMI shielding performance of a material is closely associated with the intrinsic electromagnetic properties of that material and is also dependent on other factors such as the frequency of the radiation, thickness of the material, and temperature [49,60,61]. Measured in decibels (dB), shielding effectiveness (SE) is a material’s ability to attenuate electromagnetic radiations and is the logarithmic ratio of the incident power (P_I_) to the transmitted power (P_T_) according to Equation (2). The EMI SE is the combined result of the reflection relative to the surface of the shielding material, the EM energy absorbed within the material, and the multiple scattering of EM radiation inside the material [62,63,64]. Therefore, total shielding effectiveness (SE_T_) of the shielding material can be described by the summation of the shielding effectiveness due to reflection (SE_R_), absorption (SE_A_) and multiple reflections (SE_M_) [9]. Powers reflected and transmitted can be determined from the S-parameters measured by the vector network analyser as a ratio of the incident power [65], as shown in Equations (3)–(5). The S-parameters, S_11_ and S_21_, are detected by ports 1 and 2 of the vector network analyser, respectively (Figure 2a).

(2) SEt=SER+SEA+SEM=−10log(PIPT)=10 log101|S21|2

(3) Transmissivity:PTPI=|S21|2

(4) Reflectivity:PRPI=|S11|2

(5) Absorptivity:PAPI=1−PTPI−PRPI

Figure 5 shows that pure PBAT is transparent to the microwaves; even at high frequencies of Ku-band, it exhibits SE of less than 1 dB. This is due to the very low electrical conductivity (~10^−11^ S/m), as well as low electromagnetic properties of pure PBAT. *SE_T_* of the nanocomposites increases upon increasing GNP loading due to the enhancement of electromagnetic properties of PBAT, in particular conductivity. In Figure 5a, increasing the GNP loading up to 6 wt % gradually increases the SE_T_ to about 3.4 dB. As the GNP concentration is raised from 6 to 9 wt %, a 2 dB increment is observed in the SE_T_, and from 9 to 12 wt %, an increment of about 3 dB is detected. The stepwise increase in the SE_T_ of the nanocomposites with 6 to 9 wt % GNPs can be attributed to the GNP electrical percolation observed in Figure 4a. However, from 12 to 15 wt %, the enhancement in SE_T_ slows down, exhibiting an improvement of only 1.35 dB over the C-band frequency range. Figure 5b demonstrates a trend similar to the one observed in Figure 5a for the SE_T_ behaviour of nanocomposites in the X-band versus GNP loading with the maximum of 14 dB at 15 wt % GNPs. The effect of GNP embedding on the total shielding effectiveness of PBAT can be clearly perceived from Figure 5d. As mentioned earlier, SE of a shielding material is dependent on the material’s electromagnetic properties including electrical conductivity, electrical permittivity, and magnetic permeability, as well as the radiation frequency and the material’s thickness [66]. Data reported in Figure 5 are for the same frequency range and nanocomposite thickness. Considering that graphene nanoplatelets do not have magnetic properties, the variations in the EMI SE behaviour of PBAT/GNP nanocomposites are due to the enhancement of the electrical conductivity and electrical permittivity (dielectric constant and dielectric loss) of PBAT as the GNP loading is increased.

An interesting observation from Figure 5c is that while SE_T_ increases with increasing GNP loading, it exhibits a decreasing trend versus frequency for highly filled nanocomposites of 12 and 15 wt %. Effects of radiation frequency and nanocomposite thickness will be discussed later on in the article. It is noteworthy to mention here that temperature can also be an important factor affecting the EMI SE of a shielding material due to the temperature dependence of the materials’ electromagnetic properties. This topic has been extensively studied in references [63,66,67], and different mechanisms of radiation absorption and their temperature dependence have been discussed.

**Figure 6:**
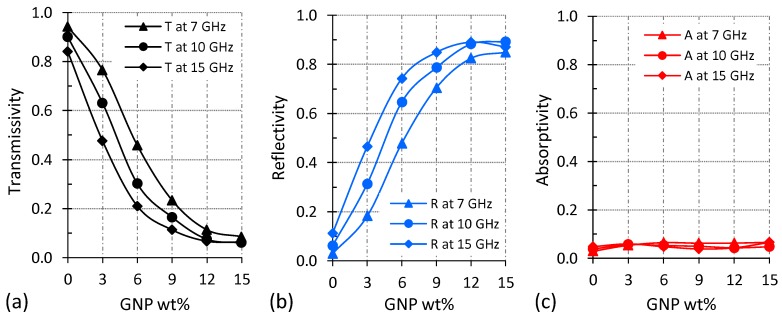
Variations of the (**a**) transmissivity, (**b**) reflectivity, and (**c**) absorptivity of PBAT/GNP nanocomposites with GNP concentration at different frequencies.

The contributions of reflection and absorption mechanisms in the overall shielding performance of the PBAT/GNP nanocomposites as functions of GNP loading and frequency are depicted in Figure 6. Transmissivity, reflectivity, and absorptivity were calculated according to Equations (3)–(5). As the GNP loading increases, reflectivity enhances, and as a result, transmissivity decreases. At mid C-band, reflectivity increases almost uniformly with increasing GNP loading up to 12 wt %, reaching a value of 0.82. However, further increase in GNP concentration from 12 to 15 wt % augments the reflectivity by only 0.05. At higher frequencies, the rate of increase in the reflectivity is markedly higher at lower GNP loadings compared to higher GNP concentrations. At mid Ku-band, reflectivity increases to 0.74 by addition of only 6 wt % GNPs, while increasing the GNP loading from 6 to 15 wt % enhances the reflectivity by just 0.13. Although enhanced, absorptivity does not show significant variations with increasing the GNP concentration (Figure 6c). However, it is worth mentioning that the absorptivity values reported in Figure 6c are those of 1 mm thick samples. Equations (6) and (7) show the parameters affecting the amount of power reflected and absorbed by a shielding material in a simplified manner. In these equations, σ is the electrical conductivity, μ is the magnetic permeability, f is the frequency and d is the thickness of the shielding material [68,69]. The direct relation between absorbed power and shielding material’s thickness can clearly be seen in Equation (7). 

(6) $${\mathrm{SE}}_{R}=39.5+10\log_{10}\frac{\sigma }{2\pi f\mu }$$

(7) $${\mathrm{SE}}_{A}=8.7d\sqrt{\pi f\mu \sigma }$$

(8) $$A_{\mathrm{effective}}=\left(\frac{\mathrm{Absorbed}\;\mathrm{power}}{1-\mathrm{Reflected}\;\mathrm{power}}\right)$$

**Figure 7:**
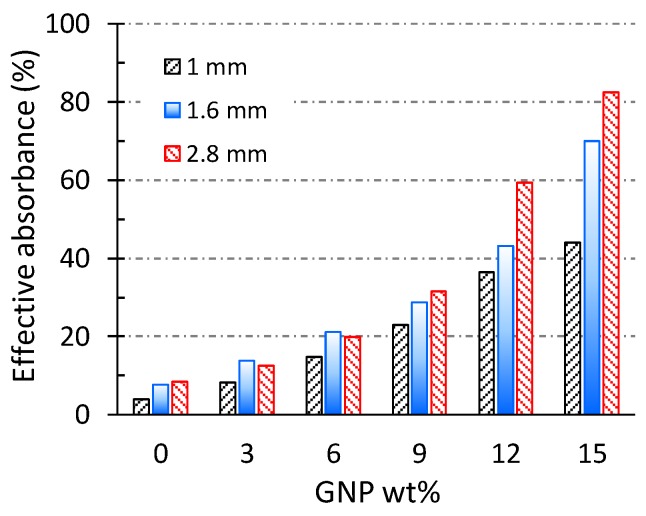
Effective absorbance of PBAT/GNP nanocomposites with different thicknesses as a function of GNP loading at mid X-band frequency (10 GHz).

In addition to the 1 mm thick samples of PBAT/GNP nanocomposites, S-parameters of samples with thicknesses of 1.5 mm and 2.8 mm were also collected over the X-band frequency range to investigate the effect of thickness on their microwave absorption potential. Effective absorbance values of these samples, calculated based on Equation 8, are reported in Figure 7. In contrast to the absorptivity (Figure 6c), which is the absolute value of the power absorbed, effective absorbance is the ratio of the power absorbed to the power which enters the sample, eliminating the effect of reflection [70]. For each PBAT/GNP composition, it is observed that A_eff_ increases with increasing thickness. In particular, for high GNP loadings, the effect of thickness on A_eff_ is more significant due to the higher electromagnetic properties of these nanocomposites. It is also interesting to note that when the actual power entering in the sample is taken into account rather than the incident power, even a thin sample of PBAT + 15 wt % GNPs absorbs more than 40% of the radiation.

**Figure 8:**
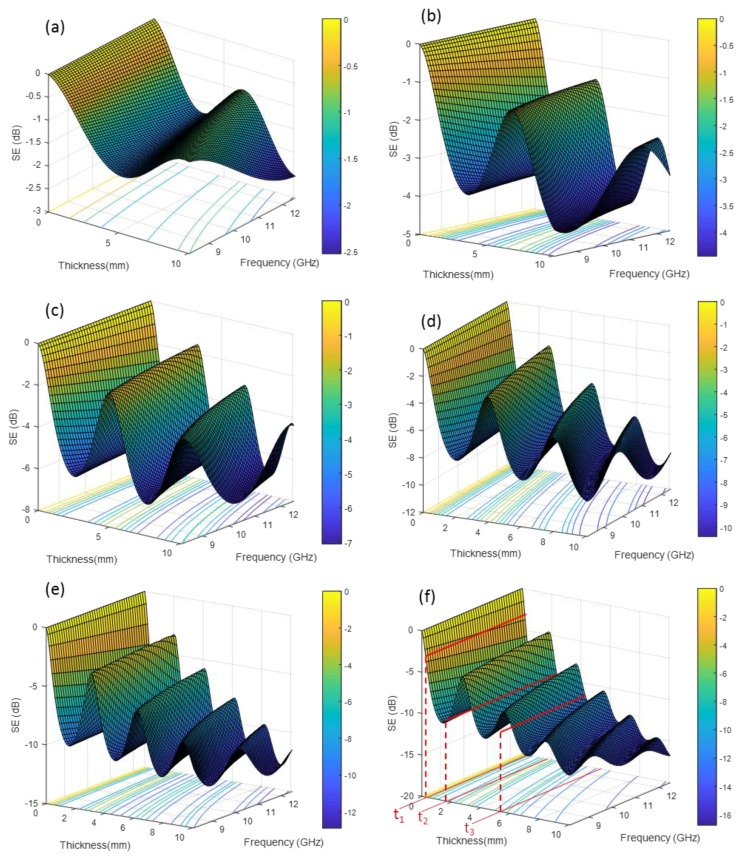
Shielding effectiveness of PBAT/GNP nanocomposites as a function of thickness and frequency for (**a**) 0, (**b**) 3, (**c**) 6, (**d**) 9, (**e**) 12, and (**f**) 15 wt % GNP content.

In Figure 5, the SE_T_ profiles of PBAT/GNP nanocomposites over the C- and X-bands do not show significant variation with frequency. On the other hand, an interesting observation in this figure is that at the higher frequencies of Ku-band, SE_T_ profiles of PBAT/GNP nanocomposites with 12 and 15 wt % show a decreasing trend with increasing frequency. In particular, SE_T_ of the nanocomposite with 15 wt % GNPs decreases markedly from 15.26 dB to 7.26 dB (almost halved) as the frequency increases from 12 GHz to 18 GHz. This behaviour can be attributed to the complicated nature of the interactions between the microwaves and the shielding material. Equations (6) and (7) show that shielding by reflection decreases with increase in the product of (*f*μ) while shielding by absorption increases [69]. To better understand the shielding performance of the PBAT/GNP nanocomposites with regard to the microwave frequency and sample thickness, SE behaviours of pure PBAT and its GNP nanocomposites over the X-band are depicted in Figure 8. Considering that electromagnetic properties of materials are intensive, electrical properties of the PBAT/GNP nanocomposites [61] at mid X-band frequency were used to calculate their S-parameters over the 8.2–12.4 GHz range for a thickness range of 0–10 mm by using Equations (9)–(12). In these equations, ω is the wave’s angular frequency, c is the wave’s speed in free space, µ_r_ and ε_r_ are the magnetic permeability and electrical permittivity of the material, respectively, relative to those of free space, and d is the sample thickness [71]. The negative values of SE in Figure 8 are a result of not using the negative sign of Equation (2).

A periodic variation in the SE_T_ behaviour of the nanocomposites with thickness is observed in Figure 8; at any fixed frequency, increasing the thickness results in sine-wave periodic changes in the SE_T_ with a general increasing trend for all the PBAT/GNP compositions. For the thickness, on the other hand, two different behaviours are observed in the SE_T_. As demonstrated in Figure 8f, for a thin sample (t_1_), SE_T_ remains almost constant over the entire frequency range but for a thicker sample it exhibits frequency dependency. For example, for thickness t_2_, SE_T_ decreases (blue to green contours) with increasing frequency, while for thickness t_3_, SE_T_ increases (green to blue contours). Another observation from Figure 8 is that as the GNP loading increases, the wave-like behaviour of SE_T_ shows more fluctuations with smaller periods. This trend can be attributed to the higher electrical properties of the nanocomposites with greater loadings of conductive graphene nanoplatelets, leading to complicated multiple reflections of the microwave inside the sample. In general, whenever the medium in which the electromagnetic waves are travelling changes, the waves will be partially reflected back at the interface of the two media. The higher the difference between the electromagnetic properties of the media (impedance mismatch), the greater the reflected power. Therefore, PBAT/GNP nanocomposites with higher GNP content will have greater impedance mismatch with air. The multiple reflections can have both destructive and constructive effects on the overall shielding performance of the nanocomposites. Consequently, when the SE_T_ is at its minimum, the multiple reflections are destructive and when the SE_T_ is at its highest value, the multiple reflections have a constructive effect on the overall shielding performance of the shielding material [72].

(9) $$\left|s_{11}\right|=\left|\frac{\left(1-z^{2}\right)\Gamma }{1-\Gamma^{2}z^{2}}\right|$$

(10) $$\left|s_{21}\right|=\left|\frac{\left(1-\Gamma^{2}\right)z}{1-\Gamma^{2}z^{2}}\right|$$

(11) $$\Gamma =\frac{\sqrt{\frac{\mu_{r}}{\epsilon_{r}}}-1}{\sqrt{\frac{\mu_{r}}{\epsilon_{r}}}+1}$$

(12) $$z=\exp\left(-j\left(\omega /c\right)\sqrt{\mu_{r}\epsilon_{r}}d\right)$$

## 4. Conclusions

The EMI shielding capability, in C-, X-, and Ku-bands, was assessed for a set of nanocomposites composed of graphene nanoplatelets and poly(butylene adipate-*co*-terephthalate) matrix. SE measurements were conducted on samples with thickness of 1 mm. GNP incorporation significantly increased the electrical conductivity of the insulating polymeric matrix, resulting in enhanced SE_T_. The highest rate of increase in SE_T_ was observed near the percolation threshold of GNPs. Comparison of reflectivity and absorptivity revealed that reflection played a major role in the shielding potential of nanocomposites for all three frequency ranges. The high amount of power reflected at the nanocomposites surface led to low absorptivity (below 0.05). However, by eliminating the effect of reflection by using effective absorbance, the true potential of the nanocomposites for absorbing microwaves was evaluated. At 15 wt % GNP loading, a 1-mm-thick sample attenuated more than 40% of the power entering the sample. Effective absorbance of the nanocomposites for two higher thicknesses showed a direct relation between the effective absorbance and the thickness with a 2.8-mm-thick sample having an absorbance potential of more than 80% at 15 wt % GNPs. The measurement also showed that the SE_T_ of nanocomposites with low GNP content did not vary appreciably with changing frequency. On the other hand, SE_T_ of highly filled nanocomposites decreased markedly with increasing frequency over Ku-band. Simulation of the SE_T_ behaviour of the nanocomposites for the X-band frequency range as a function of thickness revealed a periodic trend in SE_T_. Depending on the thickness, SE_T_ exhibited different frequency dependency trends; for thin samples, SE_T_ almost remain unchanged while for thicker samples it demonstrated both increasing and decreasing trends with increasing frequency.

## References

[1] Wallace P.R. (1947). The Band Theory of Graphite. Phys. Rev..

[2] Novoselov K.S., Geim A.K., Morozov S.V., Jiang D., Zhang Y., Dubonos S.V., Grigorieva I.V., Firsov A.A. (2004). Electric Field Effect in Atomically Thin Carbon Films. Science.

[3] Abbott’s I.E. (2007). Graphene: Exploring carbon flatland. Phys. Today.

[4] Potts J.R., Dreyer D.R., Bielawski C.W., Ruoff R.S. (2011). Graphene-based polymer nanocomposites. Polymer.

[5] Suk J.W., Piner R.D., An J., Ruoff R.S. (2010). Mechanical properties of monolayer graphene oxide. ACS Nano.

[6] Wang G., Yang J., Park J., Gou X., Wang B., Liu H., Yao J. (2008). Facile synthesis and characterization of graphene nanosheets. J. Phys. Chem. C.

[7] Mao C., Zhu Y., Jiang W. (2012). Design of electrical conductive composites: Tuning the morphology to improve the electrical properties of graphene filled immiscible polymer blends. ACS Appl. Mater. Interfaces.

[8] Mao C., Huang J., Zhu Y., Jiang W., Tang Q., Ma X. (2012). Tailored parallel graphene stripes in plastic film with conductive anisotropy by shear-induced self-assembly. J. Phys. Chem. Lett..

[9] Verma M., Chauhan S.S., Dhawan S.K., Choudhary V. (2017). Graphene nanoplatelets/carbon nanotubes/polyurethane composites as efficient shield against electromagnetic polluting radiations. Compos. Part B Eng..

[10] Kuilla T., Bhadra S., Yao D., Kim N.H., Bose S., Lee J.H. (2010). Recent advances in graphene based polymer composites. Prog. Polym. Sci..

[11] Allen M.J., Tung V.C., Kaner R.B. (2009). Honeycomb carbon: A review of graphene. Chem. Rev..

[12] Liu H., Huang W., Yang X., Dai K., Zheng G., Liu C., Shen C., Yan X., Guo J., Guo Z. (2016). Organic vapor sensing behaviors of conductive thermoplastic polyurethane–graphene nanocomposites. J. Mater. Chem. C.

[13] Liu H., Dong M., Huang W., Gao J., Dai K., Guo J., Zheng G., Liu C., Shen C., Guo Z. (2017). Lightweight conductive graphene/thermoplastic polyurethane foams with ultrahigh compressibility for piezoresistive sensing. J. Mater. Chem. C.

[14] Luo Q., Ma H., Hou Q., Li Y., Ren J., Dai X., Yao Z., Zhou Y., Xiang L., Du H. (2018). All-Carbon-Electrode-Based Endurable Flexible Perovskite Solar Cells. Adv. Funct. Mater..

[15] Kashi S., Gupta R., Kao N., Bhattacharya S. Preparation and Characterization of PolyLactide and Poly(Butylene Adipate-*co*-Terephthalate) Nanocomposites Reinforced with Graphene Nanoplatelet. Proceedings of the 40th Annual Condensed Matter and Materials Meeting.

[16] Hu Z., Shao Q., Moloney M.G., Xu X., Zhang D., Li J., Zhang C., Huang Y. (2017). Nondestructive functionalization of graphene by surface-initiated atom transfer radical polymerization: An ideal nanofiller for poly(p-phenylene benzobisoxazole) fibers. Macromolecules.

[17] Hu Z., Wang C., Zhao F., Xu X., Wang S., Yu L., Zhang D., Huang Y. (2017). Fabrication of a graphene/C 60 nanohybrid via γ-cyclodextrin host–guest chemistry for photodynamic and photothermal therapy. Nanoscale.

[18] Guo Y., Xu G., Yang X., Ruan K., Ma T., Zhang Q., Gu J., Wu Y., Liu H., Guo Z. (2018). Significantly enhanced and precisely modeled thermal conductivity in polyimide nanocomposites with chemically modified graphene via in situ polymerization and electrospinning-hot press technology. J. Mater. Chem. C.

[19] Pawar S.P., Stephen S., Bose S., Mittal V. (2015). Tailored electrical conductivity, electromagnetic shielding and thermal transport in polymeric blends with graphene sheets decorated with nickel nanoparticles. Phys. Chem. Chem. Phys..

[20] Wan Y.-J., Yu S.-H., Yang W.-H., Zhu P.-L., Sun R., Wong C.-P., Liao W.-H. (2016). Tuneable cellular-structured 3D graphene aerogel and its effect on electromagnetic interference shielding performance and mechanical properties of epoxy composites. RSC Adv..

[21] Gavgani J.N., Adelnia H., Zaarei D., Gudarzi M.M. (2016). Lightweight flexible polyurethane/reduced ultralarge graphene oxide composite foams for electromagnetic interference shielding. RSC Adv..

[22] Ma C.C.M., Huang Y.L., Kuan H.C., Chiu Y.S. (2005). Preparation and electromagnetic interference shielding characteristics of novel carbon-nanotube/siloxane/poly-(urea urethane) nanocomposites. J. Polym. Sci. Part B Polym. Phys..

[23] Wu H.L., Ma C.C.M., Yang Y.T., Kuan H.C., Yang C.C., Chiang C.L. (2006). Morphology, electrical resistance, electromagnetic interference shielding and mechanical properties of functionalized MWNT and poly(urea urethane) nanocomposites. J. Polym. Sci. Part B Polym. Phys..

[24] Nimbalkar P., Korde A., Goyal R.K. (2018). Electromagnetic interference shielding of polycarbonate/GNP nanocomposites in X-band. Mater. Chem. Phys..

[25] Wan Y.-J., Zhu P.-L., Yu S.-H., Sun R., Wong C.-P., Liao W.-H. (2017). Ultralight, super-elastic and volume-preserving cellulose fiber/graphene aerogel for high-performance electromagnetic interference shielding. Carbon.

[26] Wan Y.-J., Zhu P.-L., Yu S.-H., Sun R., Wong C.-P., Liao W.-H. (2017). Graphene paper for exceptional EMI shielding performance using large-sized graphene oxide sheets and doping strategy. Carbon.

[27] Luo C., Duan W., Yin X., Kong J. (2016). Microwave-absorbing polymer-derived ceramics from cobalt-coordinated poly(dimethylsilylene) diacetylenes. J. Phys. Chem. C.

[28] Zhang K., Li G.-H., Feng L.-M., Wang N., Guo J., Sun K., Yu K.-X., Zeng J.-B., Li T., Guo Z. (2017). Ultralow percolation threshold and enhanced electromagnetic interference shielding in poly(l-lactide)/multi-walled carbon nanotube nanocomposites with electrically conductive segregated networks. J. Mater. Chem. C.

[29] Song Y., He L., Zhang X., Liu F., Tian N., Tang Y., Kong J. (2017). Highly Efficient Electromagnetic Wave Absorbing Metal-Free and Carbon-Rich Ceramics Derived from Hyperbranched Polycarbosilazanes. J. Phys. Chem. C.

[30] Liu J., Zhang H.-B., Liu Y., Wang Q., Liu Z., Mai Y.-W., Yu Z.-Z. (2017). Magnetic, electrically conductive and lightweight graphene/iron pentacarbonyl porous films enhanced with chitosan for highly efficient broadband electromagnetic interference shielding. Compos. Sci. Technol..

[31] Yang H., Yu Z., Wu P., Zou H., Liu P. (2018). Electromagnetic interference shielding effectiveness of microcellular polyimide/in situ thermally reduced graphene oxide/carbon nanotubes nanocomposites. Appl. Surf. Sci..

[32] Bi S., Zhang L., Mu C., Lee H.Y., Cheah J.W., Chua E.K., See K.Y., Liu M., Hu X. (2017). A comparative study on electromagnetic interference shielding behaviors of chemically reduced and thermally reduced graphene aerogels. J. Colloid Interface Sci..

[33] Cui C.-H., Yan D.-X., Pang H., Jia L.-C., Bao Y., Jiang X., Li Z.-M. (2016). Towards efficient electromagnetic interference shielding performance for polyethylene composites by structuring segregated carbon black/graphite networks. Chin. J. Polym. Sci..

[34] Lu Z., Ma L., Tan J., Wang H., Ding X. (2016). Transparent multi-layer graphene/polyethylene terephthalate structures with excellent microwave absorption and electromagnetic interference shielding performance. Nanoscale.

[35] Chen Z., Xu C., Ma C., Ren W., Cheng H.M. (2013). Lightweight and flexible graphene foam composites for high-performance electromagnetic interference shielding. Adv. Mater..

[36] Zhang H.-B., Yan Q., Zheng W.-G., He Z., Yu Z.-Z. (2011). Tough graphene−polymer microcellular foams for electromagnetic interference shielding. ACS Appl. Mater. Interfaces.

[37] Yu H., Wang T., Wen B., Lu M., Xu Z., Zhu C., Chen Y., Xue X., Sun C., Cao M. (2012). Graphene/polyaniline nanorod arrays: Synthesis and excellent electromagnetic absorption properties. J. Mater. Chem..

[38] Song W.-L., Cao M.-S., Lu M.-M., Bi S., Wang C.-Y., Liu J., Yuan J., Fan L.-Z. (2014). Flexible graphene/polymer composite films in sandwich structures for effective electromagnetic interference shielding. Carbon.

[39] Liang J., Wang Y., Huang Y., Ma Y., Liu Z., Cai J., Zhang C., Gao H., Chen Y. (2009). Electromagnetic interference shielding of graphene/epoxy composites. Carbon.

[40] Kausar A., Anwar Z., Khan L.A., Muhammad B. (2016). Functional graphene nanoplatelet reinforced epoxy resin and polystyrene-based block copolymer nanocomposite. Fuller. Nanotub. Carbon Nanostruct..

[41] Sun X., Liu X., Shen X., Wu Y., Wang Z., Kim J.-K. (2016). Graphene foam/carbon nanotube/poly(dimethyl siloxane) composites for exceptional microwave shielding. Compos. Part A Appl. Sci. Manuf..

[42] Cao M.-S., Wang X.-X., Cao W.-Q., Yuan J. (2015). Ultrathin graphene: Electrical properties and highly efficient electromagnetic interference shielding. J. Mater. Chem. C.

[43] Cao M., Han C., Wang X., Zhang M., Zhang Y., Shu J., Yang H., Fang X., Yuan J. (2018). Graphene nanohybrids: Excellent electromagnetic properties for the absorbing and shielding of electromagnetic waves. J. Mater. Chem. C.

[44] Cao W.-Q., Wang X.-X., Yuan J., Wang W.-Z., Cao M.-S. (2015). Temperature dependent microwave absorption of ultrathin graphene composites. J. Mater. Chem. C.

[45] Lu M., Wang X., Cao W., Yuan J., Cao M. (2016). Carbon nanotube-CdS core-shell nanowires with tunable and high-efficiency microwave absorption at elevated temperature. Nanotechnology.

[46] Wang X.-X., Ma T., Shu J.-C., Cao M.-S. (2018). Confinedly tailoring Fe_3_O_4_ clusters-NG to tune electromagnetic parameters and microwave absorption with broadened bandwidth. Chem. Eng. J..

[47] Cao M.-S., Yang J., Song W.-L., Zhang D.-Q., Wen B., Jin H.-B., Hou Z.-L., Yuan J. (2012). Ferroferric oxide/multiwalled carbon nanotube vs. polyaniline/ferroferric oxide/multiwalled carbon nanotube multiheterostructures for highly effective microwave absorption. ACS Appl. Mater. Interfaces.

[48] Zhang Y., Wang X., Cao M. (2018). Confinedly implanted NiFe_2_O_4_-rGO: Cluster tailoring and highly tunable electromagnetic properties for selective-frequency microwave absorption. Nano Res..

[49] Kashi S., Gupta R.K., Baum T., Kao N., Bhattacharya S.N. (2016). Morphology, electromagnetic properties and electromagnetic interference shielding performance of polylactide/graphene nanoplatelet nanocomposites. Mater. Des..

[50] Kashi S., Gupta R.K., Kao N., Hadigheh S.A., Bhattacharya S.N. (2017). Influence of graphene nanoplatelet incorporation and dispersion state on thermal, mechanical and electrical properties of biodegradable matrices. J. Mater. Sci. Technol..

[51] Ecoflex F Blend C1200 Data Sheet. http://www.plasticsportal.net/wa/plasticsEU~tr_TR/function/conversions:/publish/common/upload/biodegradable_plastics/Ecoflex_F_Blend_C1200.pdf.

[52] Technical Data Sheet-xGnP® Graphene Nanoplatelets Grade M Characteristics. https://xgsciences.com/wp-content/uploads/2017/11/xGnP-M.-MD00003.-2018-1.pdf.

[53] Kashi S., Gupta R.K., Kao N., Bhattacharya S.N. (2016). Electrical, thermal, and viscoelastic properties of graphene nanoplatelet/poly(butylene adipate-*co*-terephthalate) biodegradable nanocomposites. J. Appl. Polym. Sci..

[54] Kashi S., Gupta R.K., Baum T., Kao N., Bhattacharya S.N. (2018). Phase transition and anomalous rheological behaviour of polylactide/graphene nanocomposites. Compos. Part B Eng..

[55] Barrau S., Demont P., Peigney A., Laurent C., Lacabanne C. (2003). DC and AC Conductivity of Carbon Nanotubes−Polyepoxy Composites. Macromolecules.

[56] Al-Saleh M.H., Sundararaj U. (2012). Microstructure, electrical, and electromagnetic interference shielding properties of carbon nanotube/acrylonitrile–butadiene–styrene nanocomposites. J. Polym. Sci. Part B Polym. Phys..

[57] Bhattacharya S.N., Kamal M.R., Gupta R.K. (2008). Polymeric Nanocomposites: Theory and Practice.

[58] Kashi S., Gupta R.K., Kao N., Bhattacharya S.N. (2017). Rheology and physical characterization of graphene nanoplatelet/poly(butylene adipate-*co*-terephthalate) nanocomposites. AIP Conference Proceedings.

[59] Kashi S., Gupta R.K., Kao N., Bhattacharya S.N. (2016). Viscoelastic properties and physical gelation of poly(butylene adipate-*co*-terephthalate)/graphene nanoplatelet nanocomposites at elevated temperatures. Polymer.

[60] Zhao B., Zhao C., Li R., Hamidinejad S.M., Park C.B. (2017). Flexible, Ultrathin, and High-Efficiency Electromagnetic Shielding Properties of Poly(Vinylidene Fluoride)/Carbon Composite Films. ACS Appl. Mater. Interfaces.

[61] Kashi S., Gupta R.K., Baum T., Kao N., Bhattacharya S.N. (2016). Dielectric properties and electromagnetic interference shielding effectiveness of graphene-based biodegradable nanocomposites. Mater. Des..

[62] Wen B., Cao M.-S., Hou Z.-L., Song W.-L., Zhang L., Lu M.-M., Jin H.-B., Fang X.-Y., Wang W.-Z., Yuan J. (2013). Temperature dependent microwave attenuation behavior for carbon-nanotube/silica composites. Carbon.

[63] Cao M.-S., Song W.-L., Hou Z.-L., Wen B., Yuan J. (2010). The effects of temperature and frequency on the dielectric properties, electromagnetic interference shielding and microwave-absorption of short carbon fiber/silica composites. Carbon.

[64] Cao M.-S., Wang X.-X., Cao W.-Q., Fang X., Wen B., Yuan J. (2018). Thermally-Driven Transport and Relaxation Switching Self-powered Electromagnetic Energy Conversion. Small.

[65] Chhetri S., Samanta P., Chandra Murmu N., Kumar Srivastava S., Kuila T. (2016). Electromagnetic interference shielding and thermal properties of non-covalently functionalized reduced graphene oxide/epoxy composites. AIMS Mater. Sci..

[66] Wen B., Cao M., Lu M., Cao W., Shi H., Liu J., Wang X., Jin H., Fang X., Wang W. (2014). Reduced graphene oxides: Light-weight and high-efficiency electromagnetic interference shielding at elevated temperatures. Adv. Mater..

[67] Song W.-L., Cao M.-S., Hou Z.-L., Fang X.-Y., Shi X.-L., Yuan J. (2009). High dielectric loss and its monotonic dependence of conducting-dominated multiwalled carbon nanotubes/silica nanocomposite on temperature ranging from 373 to 873 K in X-band. Appl. Phys. Lett..

[68] Al-Saleh M.H. (2016). Electrical, EMI shielding and tensile properties of PP/PE blends filled with GNP:CNT hybrid nanofiller. Synth. Met..

[69] Al-Saleh M.H., Sundararaj U. (2009). Electromagnetic interference shielding mechanisms of CNT/polymer composites. Carbon.

[70] Bansala T., Joshi M., Mukhopadhyay S., Doong R.-A., Chaudhary M. (2016). Electrically conducting graphene-based polyurethane nanocomposites for microwave shielding applications in the Ku band. J. Mater. Sci..

[71] Nicolson A.M., Ross G.F. (1970). Measurement of the Intrinsic Properties of Materials by Time-Domain Techniques. IEEE Trans. Instrum. Meas..

[72] Amiet A. (2003). Free Space Permittivity and Permeability Measurements at Microwave Frequencies.

